# The Oceanic Biological Pump: Rapid carbon transfer to depth at Continental Margins during Winter

**DOI:** 10.1038/s41598-017-11075-6

**Published:** 2017-09-07

**Authors:** Laurenz Thomsen, Jacopo Aguzzi, Corrado Costa, Fabio De Leo, Andrea Ogston, Autun Purser

**Affiliations:** 1 Jacobs University, Bremen, 28759 Germany; 2 Instituto de Ciencias del Mar (ICM-CSIC), Barcelona, 08003 Spain; 3 Consiglio per la ricerca in agricoltura e l’analisi dell’economina agrarian (CREA-IT), Monterotondo, 00016 Italy; 4 Ocean Networks Canada and Department of Biology, University of Victoria, Victoria, BC V8W 2Y2 Canada; 5 School of Oceanography, University of Washington, Seattle, 98195 USA; 6 Alfred Wegener Institute for Polar Research, Bremerhaven, 27570 Germany

## Abstract

The oceanic biological pump is responsible for the important transfer of CO_2_-C as POC “Particulate Organic Carbon” to the deep sea. It plays a decisive role in the Earth’s carbon cycle and significant effort is spent to quantify its strength. In this study we used synchronized daily time-series data of surface chlorophyll-a concentrations from the NASA’s MODIS satellite in combination with hourly to daily observations from sea surface buoys and from an Internet Operated Vehicle (IOV) on the seafloor within Barkley Canyon (Northeast Pacific) to investigate the importance of winter processes in the export of fresh phytodetritus. The results indicate that phytoplankton pulses during winter can be as important in POC transfer to depth as the pulses associated with spring and summer blooms. Short winter phytoplankton pulses were observed to disappear from surface waters after low-pressure systems affected the area. Pulses of chlorophyll reached the IOV, at 870 m depth on the canyon seafloor, 12–72 hours later. These observed short pulses of biological carbon production regularly observed in the region from December to March have not been considered a significant component of the biological pump when compared with the denser summer productivity blooms.

## Introduction

**Figure 1:**
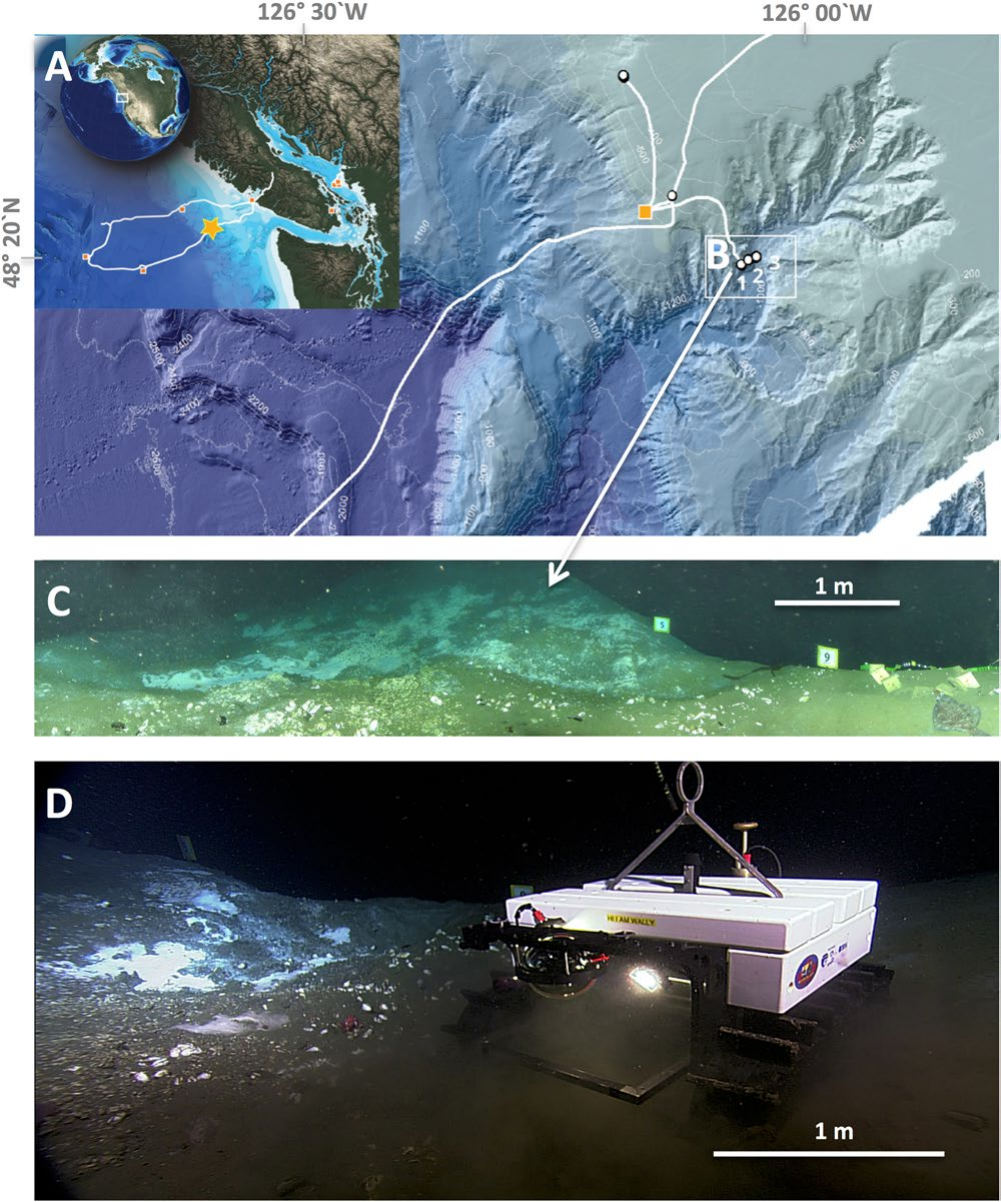
Map and photographs of ONC study site at 870 m water depth in Barkley Canyon off Vancouver Island, British Columbia. The figure shows the ONC network (**A**) and the study site (B1,**C**,**D**) on a small plateau in the canyon, located approximately 1 km away from canyon axis (B3). Distance to 400 m water depth via the canyon flanks is 3 km. Several canyon heads at 300-m depth are 5–8 km away. Approximately 20 m^2^ of seafloor were frequently monitored with a sensor-equipped Internet Operated Vehicle (IOV) from November 2010 to June 2011 (**D**). Despite the complex topography of the canyon, flow velocity measurements with ADCPs at both the canyon axis (B3) and on the flanks (B2) exhibited a relatively persistent downslope component of 0.047 ± 0.017 m s^−1^ to the flow throughout the winter period. Data used in this work were provided by Ocean Networks Canada.

A general concept of the oceanic biological pump implies that up to 90% of the annual export of particulate organic carbon (POC) occurs during a few episodic pulses throughout the year, following strong seasonal phytoplankton blooms, and that these pulse signals gradually diminish in magnitude with depth via progressive decomposition^1^. The influence of highly productive continental margins on deep ocean organic carbon inventories is increasingly represented in global carbon models^2^, with this biological pump being the core subject of several international multidisciplinary research programs conducted over the last 30 years^3–5^. Submarine canyons along these margins have been identified as potential rapid conduits for POC to the deep ocean via lateral advection, but detailed long-term observations on how these fluxes may be funneled through canyons are few^6^, despite the fact that there are approximately 9,500 canyons in the world’s oceans^7^. Even fewer studies have examined the importance of winter phytoplankton productivity and transport processes in this export^8–12^. Evaluating carbon export flux to the deep sea has traditionally relied on the collection of data and physical samples using oceanographic vessels, sediment traps, and autonomous landers^1, 6^. Here we used a combination of satellite imagery and data from a sensor-equipped Internet Operated Vehicle (IOV) connected to the Ocean Networks Canada (ONC) cabled observatory at depth within Barkley Canyon for time-series observations on bentho-pelagic coupling^13^ (Fig. 1). For winter 2010/2011 we concentrated our analyses on the temporal and spatial variability of the fate of short pulses of chlorophyll-a (a surrogate for fresh phytoplankton biomass) from surface waters, and its transfer through the benthic boundary layer (BBL). We present a conceptual model that links winter-driven physical forcing to small phytoplankton blooms and export to the deep sea through a canyon. Specifically, we hypothesize that intermittent mixing, and a resulting oscillation in the mixed layer depth, prior to strong seasonal stratification and the spring bloom, result in pulsed export events of POC to depth. These have a similar effect on the biological pump when compared to spring/summer conditions. These results indicate that the potential importance of winter surface productivity pulses to deep-sea ecosystems should be reconsidered.

## Results

### Analyses of weekly and daily data

For an annual overview on surface productivity, we followed the seasonal distribution of chlorophyll-a at the sea surface from January 2010 to December 2011 using 8-day composite images provided by the NASA-MODIS satellite for the continental margin off southwest Vancouver Island^14^. Data gaps resulting from cloud coverage were interpolated. Our analyses for the period October 2010 to June 2011 showed good agreement with the 8-day composites from NASA (r^2^ = 0.76). During winter and early spring several short phytoplankton pulses were detected. Measurements of chlorophyll-*a* in bottom waters of Barkley Canyon at 870-m water depth using the IOV highlight the occurrence of a considerable flux of chlorophyll-*a* during that time.

**Figure 2:**
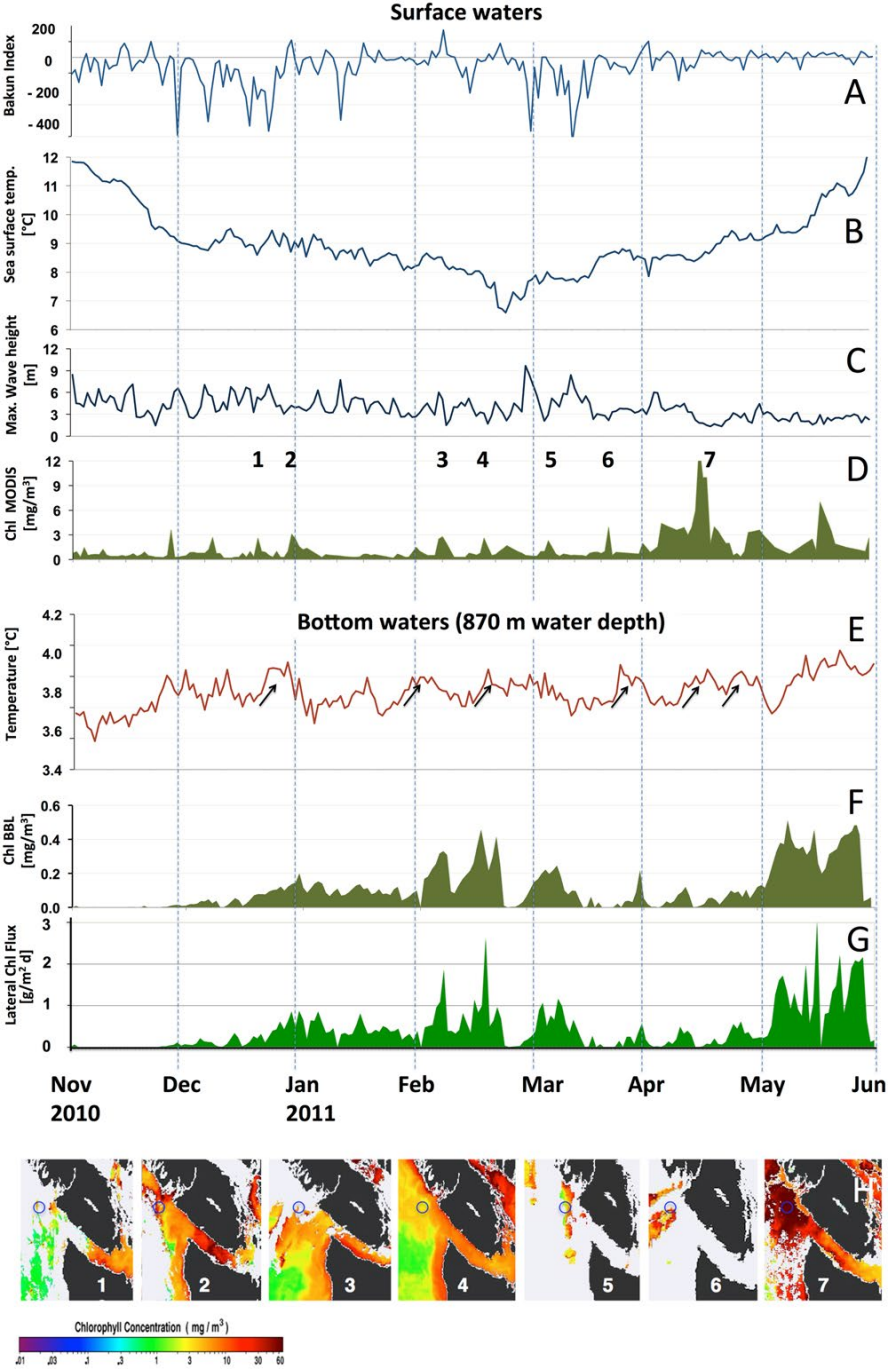
Temporal and spatial variability of oceanographic parameters and chlorophyll-*a* from daily data in both surface waters and within the BBL within Barkley Canyon. Parameters at sea surface are: (**A**) Bakun Upwelling Index from 48 N, 125°W; (**B**) Sea surface temperature; (**C**) Maximum wave height (B, C from South Brooks Buoy, Environment Canada); (**D**) chlorophyll-*a* concentration, derived from MODIS satellite data; Parameters in the BBL as measured by an IOV at 870-m depth are: (**E**) temperature with arrows indicating temperature rises associated with downslope flow, (**F**) chlorophyll-*a* concentration derived from fluorometer sensor; (**G**) Lateral fluxes of chlorophyll-a in the BBL (calculated via IOV data of flowmeter); (**H**) Satellite pictures of “Regime 3”^14^ showing phytoplankton pulses^15^ (1–7 marked in Fig. 2D) and the study site (blue circles). It is hypothesized to link the low chlorophyll concentrations in the BBL during autumn to stratification, when maximum grazer pressure and elevated SST prevented significant carbon fluxes (see also Supplementary Fig. 5).

From November to April poleward-directed winds (from 181 ± 62°) consistent with the poleward-flowing seasonal structure of the California Current System (CCS) favored downwelling and relaxation (Fig. 2A) along the coast of Vancouver Island. In early April, the wind direction turned eastward (from 232 ± 65°), weakened, and spring conditions in the CCS resulted in equatorward flows. Sea surface temperature (SST) and maximum wave height (MWH) also showed strong seasonal trends. SST steadily decreased from 12 to 7 °C between November to March, followed by an increase to 12 °C by the end of May. Average winter SST of 8.8 ± 1.1 °C showed regularly occurring rapid increases of >0.3 °C (Fig. 2B) coupled with short periods of calm weather, accompanied by short phytoplankton blooms of a few days duration. These short chlorophyll-*a* pulses, of up to 3.9 mg m^−3^, were regularly interrupted by periods of surface water cooling, accompanied by increasing MWH (≤9.6 m, Fig. 2C) during and after the passage of low pressure systems or *via* incoming swells. The phytoplankton pulses disappeared over periods of hours up to 3 days following the onset of these weather conditions in the area (Kendall-Tau −0.3, *p* < 0.0001, n = 212). At the beginning of April, when SST had returned to values ~9 °C and MWH of <4 m (Fig. 2C), surface chlorophyll-*a* concentrations reached values >3 mg m^−3^ for a period of ≈20 days (Fig. 2D), with an intense phytoplankton spring bloom of 14 mg m^−3^ chlorophyll-*a* developing April 16–23. (see Fig. 2H).

Bottom currents in the BBL were dominated by semi-diurnal tidal currents (M2 ~ 12 hrs), diurnal tidal currents (K1 ~ 24 hrs), and wind-forced near-inertial motions (13–16 hrs). These fluctuations occurred on top of weak residual downslope currents, and were accompanied by changes in near-bed temperature, which varied between 3.5–4.3 °C (Fig. 2E). The arrival of chlorophyll-a (BBL-Chl) pulses of 0–0.5 mg m^−3^ (Fig. 2F) corresponded to concentrations of up to 69% of the respective surface concentrations observed 1–3 days previously. Lag correlations between the disappearance of chlorophyll*-a* surface concentration peaks and the arrival of the peak signal at 870 m 1–3 days later were in the range of Tau = 0.15–0.17 (*p* < 0.0006–0.013, n = 212) for the daily data (see Fig. 2C,F). This BBL response to the surface signal was apparent throughout the full period of investigation, often correlated with elevated bottom water temperatures (χ^2^ test, *p* < 0.0001) indicating downwelling of slightly warmer bottom water from canyon locations further upslope, laterally advecting chlorophyll enriched water from these shallower depths to the study site (Fig. 2E). This correlation was detectable for all surface peaks except for the spring-bloom in mid-April.

### Analyses of hourly data

The daily observations of surface vs. deep waters were further investigated with waveform analyses and time-lagged canonical correlation analyses (CCA) on hourly data to evaluate temporal relationships between the cyclic fluctuation of BBL-Chl and environmental parameters observed both at the sea surface and within the canyon. CCA indicated highly significant (p < 0.0001) time lags of 12 to 72 hours between *i*. the cyclic fluctuation of an elevated chlorophyll-a signal in the BBL and *ii*. signals from both the sea surface (occurrence of low atmospheric pressure, wave height (MWH), cooling of surface waters (SST) and the BBL (temperature, tidal signal (pressure), flow-velocity) during the period of investigation (November – May, Table 1). Correlations at time lags of 12 to 72 hours between elevated BBL-Chl associated with downslope-directed flow at low tide (i.e., low BBL-pressure) and increased BBL-temperatures (i.e. downwelling events) were also detected between February and May.

**Table 1:** Time Lag (TL) Canonical Correlation Analyses (CCA) on hourly data showing only the significant temporal relationships (***highly significant, *p* < 0.0001; **significant, *p* < 0.001) between the cyclic fluctuation of the chlorophyll-a signal in the benthic boundary layer (BBL-Chl) and the environmental parameters at the sea surface and at 870 m depth. Non-significant results are not shown.

Time Lag		BBL Temp.	BBL-pressure	BBL-flow	Wave height	Atmospheric pressure	Sea Surface Temperature		CCA Coeff
Hours									
12	Nov.				0.46		−0.45	***	0.47
24	Nov.				0.61			***	0.54
36	Nov.				0.63		−0.63	***	0.76
48	Nov.				0.59		−0.55	***	0.68
60	Nov.						−0.56	***	0.68
72	Nov.						−0.68	***	0.69
12	Dec.					−0.78		***	0.76
24	Dec.				0.77	−0.83		***	0.72
36	Dec.				0.66	−0.78		***	0.79
48	Dec.				0.54	−0.87		***	0.77
60	Dec.				0.61	−0.80		***	0.71
72	Dec.					−0.67		***	0.71
12	Jan.				0.64			***	0.92
48	Jan.				−0.61		−0.53	**	0.82
60	Jan.				−0.81		−0.62	***	0.82
72	Jan.				−0.92		−0.68	***	0.97
12	Feb.	0.68	−0.73				−0.74	**	0.75
72	March	*0*.*93*					−0.73	***	0.41
24	April	0.53		−0.77				***	0.53
36	April			−0.64				***	0.46
48	April		−0.48					***	0.42
60	April		0.58					***	0.42
12	May					−0.63		***	0.70
24	May					−0.58		***	0.69
36	May	0.53				−0.50		***	0.68
48	May	0.55						***	0.67
60	May	0.66						***	0.64

**Figure 3:**
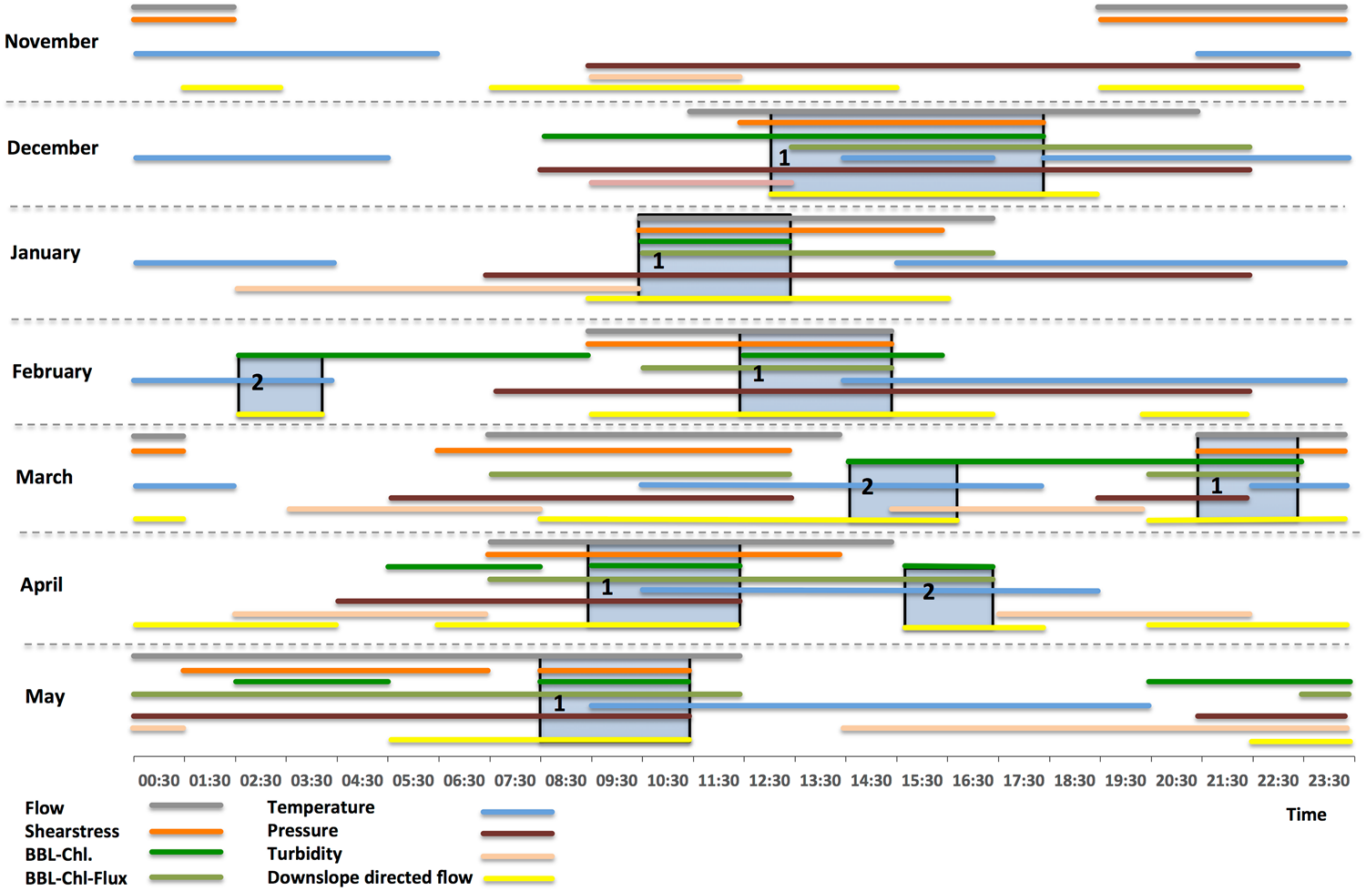
Results from monthly-based waveform analysis of 8 parameters (colored lines) using hourly data showing two reoccurring modes of downslope-transport of chlorophyll*-a* in the BBL of Barkley Canyon. The horizontal lines represent periods of the diel cycle when the parameters listed in the figure legend showed significantly increased values. The yellow horizontal line shows periods, when the flow was directed downslope. The blue shaded columns with numbers 1 and 2 represent periods of the diel cycles, when these modes occurred: Mode 1, during times of elevated flow and bottom shear stress around high tide at relatively low or only slightly elevated BBL-temperatures; and Mode 2, during times of elevated bottom water temperatures and low flow during low tide (no elevated pressure).

The waveform analyses revealed different modes of diel high-frequency (i.e. 24-h based) transferences that were typified into two major reoccurring categories (Fig. 3). Mode 1 (December to May) was characterized by downslope transport of significantly elevated BBL-Chl. These elevated fluxes coincided with elevated tidal flows and bed shear-stress, at relatively low or only slightly elevated BBL-temperatures. Mode 2 (February to April) was characterized by downslope transport of significantly elevated BBL-Chl under low flow conditions coincident with low tidal elevation, which coincided with significantly elevated BBL-temperatures. In April, when BBL-Chl remained at medium to low concentrations and SST in surface waters increased steadily during times of reduced wave height, no significant correlation with surface processes at any lag was detected, despite the intense phytoplankton spring bloom. Elevated BBL-Chl signals were only slightly correlated at a delay of 1 day with the overall Bakun up/downwelling Index (*p* < 0.01, CCA 0.06) obtained from a NOAA/NMFS/PFEG buoy located 90 km away.

## Discussion

### Conceptual model linking winter-driven physical forcing to small phytoplankton blooms and subsequent export to the deep sea at continental margins with canyons

The general concept of the oceanic biological pump assumes that the majority of the annual POC export occurs in response to strong summer phytoplankton blooms, and that winter fluxes of POC are less important or irrelevant^1^. However, as a result of applying new sampling technologies it becomes increasingly evident, that short-term, weather-induced hydrodynamic variability in the surface mixed layer can have a large impact on phytoplankton biomass, and export of primary production^12, 13^. Winter studies on the episodic and short-term POC export triggered by pulsed destratification/ stratification conditions resulting in a rapid (1–2 d) doubling of euphotic zone chlorophyll-*a* are rare, either because research vessels seldom operate in these periods of the year, or because sediment traps resolve this flux only with a resolution of weeks or months^11, 16^. A recent publication on the Santa Barbara basin adds new information, showing strong carbon fluxes to depths >140 m in January when surface production was low^12^. More detailed studies on the source/sink areas of POC are therefore clearly needed, specifically from the productive continental margins^2, 6, 9^.

**Figure 4:**
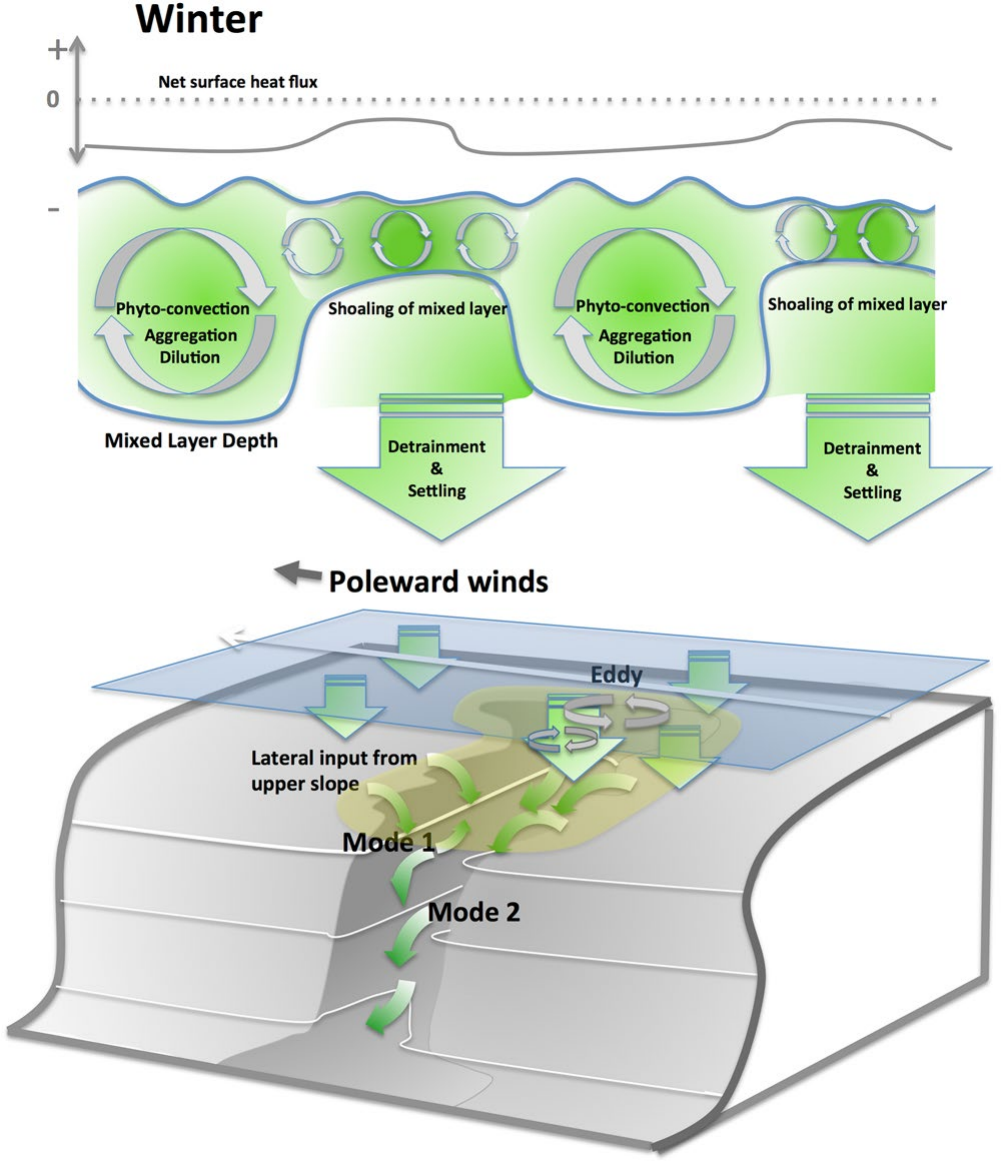
Modified model of physical and biological controls and their impacts on the winter fluxes of organic carbon through Barkley Canyon. Phyto-convection through the deepened mixed layer dilutes phytoplankton and grazers during winter, thus decoupling grazing pressure from phytoplankton growth. This leads to an increasing standing stock and subsequent phytoplankton pulses during short periods of quiescence and temperature increase with subsequent shoaling of the mixed layer. The subsequent weather induced turbulent event in surface waters causes aggregation and a detrainment of phytoplankton below the receding convective layer, from where it settles towards the seafloor and into the canyon. There, lateral transport Modes 1 and 2 (see Fig. 3) transfer the phytodetritus to depth.

We conclude from our analyses of weekly to hourly data that episodic and mainly locally occurring processes involving the surface mixed layer induced the transfer of phytoplankton along the many gullies and canyon-heads of Barkley Canyon. We expand this modified model of winter phytoplankton dynamics by also considering the interplay between wind mixing and the shutdown of deep convection at the onset of short surface blooms^17^, and the decoupling of grazer pressure via dilution of phytoplankton^18^. We infer that the POC flux to depth is mainly initiated as a detrainment process^18^ (Fig. 4), resulting from oscillations of turbulence and temperature in the mixed layer depth during winter, prior to seasonal summer stratification and the spring bloom^11^. At the location investigated in the current study, this process commences in December, at a time at which sea surface temperature has decreased to winter levels (8.8 ± 1.1 °C) and phyto-convection^17^ through the deepened turbulent mixed layer has diluted the phytoplankton and grazers, thus decoupling grazing pressure on phytoplankton by decreasing the probability of grazers finding phytoplankton efficiently^19^. The temporary decoupling leads to an increasing standing stock and a pulsed accumulation of chlorophyll biomass during the periods of quiescence, with rapid temperature increases of >0.3 °C in surface waters and subsequent shoaling of the mixed layer^20^ both factors contributing to the triggering of short duration phytoplankton production pulses^21^. These production pulses are then terminated through the subsequent phyto-convection process (turbulent mixing and resultant cooling of surface waters) which causes a detrainment of aggregated phytoplankton cells below the receding convective layer. From there these cells settle as fresh POC through the intensive remineralization zone of the upper 300 m^10^, towards the seafloor, potentially with lateral transport and further aggregation^22^ downslope. This transport driven by flows related to the larger-scale CCS circulation, the canyon morphology, tidal forcing (Fig. 3, Mode 1) and/or locally wind-forced downwelling events (Fig. 3, Mode 2). Flows at the canyon axis and on the small plateau 1 km distant, where bottom water BBL-Chl signal were measured, exhibited a relatively persistent downslope component to the flow throughout the winter period. The magnitude of the persistent downslope flow was characterized with the mean and standard deviation of the low-pass filtered currents at the canyon axis (0.047 ± 0.017 m s^−1^ in the downslope direction). In this case, the hourly current measurements were first filtered at 38 hours to remove tidal fluctuations (see also Fig. 4 of supplementary materials). At both sites, down-axis directed ebbing currents are stronger than flooding currents, with net BBL-Chl fluxes peaking during the Feb-Mar period associated with winter surface blooms. The ultimate driver of the down-canyon transfer mechanism could potentially be the bathymetric steering of seasonal current patterns and topographically induced eddies, directing surface waters into the canyons and gullies^23, 24^. These “rim eddies” are mainly cyclonic^25^ but can also develop strong anticyclonic vorticity^25, 26^ in the canyon head or anticyclonic eddies further offshore from the wider canyons in reasonable proximity^25^. Both processes can increase the vertical POC flux through a “wineglass effect”^27^ into the canyon. The lateral export process is most effective for rapidly settling diatoms, which are abundant in the area during winter^28, 29^. We estimate that the catchment area for a local vertical detrainment flux into the canyon has a size of approximately 270 km^2^ and directly surrounds the complex bathymetry of the upper canyon with its many gullies penetrating the shelf and upper slope (Fig. 1). The settling velocities of the phytoplankton entering the canyon system via the flanks (≈300–400 m deep) and canyon heads (≈300 m deep) are expected to vary between 1.2–3.4 × 10^−3^ m s^−1^ to account for the calculated delay times, thus being in the range on 1.2–8.1 × 10^−3^ m s^−1^ recently described for winter fluxes in other regions^10, 11^. The phytodetritus is then further redistributed in Barkley Canyon through local resuspension events under elevated flow, and subsequently increases its settling velocity through scavenging of denser lithogenic fraction within the BBL^22^. By this process, the transfer of fresh organic matter *via* the canyon below the intensive remineralization zone^10^ can occur within hours, since the productive shallow shelf edge- and upper slope regions are only a few kilometers away from the study site within the canyon. Even under potentially relatively low residual down-axis flow velocities of ≈0.05 m s^−1^, the fresh organic matter could reach sites of 870-m depth from different catchment areas within the canyon in 12–72 hours. The benthic community reacts to this import mainly during times of Mode 2 flow (February to April), periods characterized by downslope transport of significantly elevated BBL-Chl under low flow conditions around low tidal elevation, which coincided with significantly elevated BBL-temperatures (see supplementary materials). This indicates downwelling and down-canyon tidal pumping of water masses from shallower depths in the canyon, and associated slightly elevated temperature. With these water masses, fresh phytoplankton is observed and the signal varies at tidal frequencies^24, 26^.

The fluxes of carbon to depth surrounding the winter period of December to April can be related to the water-column stratification, where maximum grazer pressure and/or elevated SST prevent fast carbon fluxes to depth^17, 20^ since no significant correlation to surface processes at any lag was detected, despite the intense phytoplankton spring bloom. This rather results in delayed fluxes of several weeks to months, as reported for mid-mesopelagic zones in summer autumn^30^.

A lateral flux of BBL-Chl can be calculated based on observations of nearbed currents and BBL-Chl concentrations, illustrating the importance of winter conditions to downslope transport of phytodetritus (Table 2). Using MODIS data, the corresponding POC fluxes were derived from chlorophyll-a data using SeaDAS^31^, since the half-life of chlorophyll-*a* is ≈23 days^32^ and the temporal delays between the surface MODIS signals and those at 870-m depth in the canyon were only 1–3 days. The effective cumulative lateral down-canyon transport of POC through the study site over the 7-month period from November to May was estimated to be 14.5 kg m^−2^ (Mode 1 and 2), with 9.4 kg m^−2^ (65% of total flux) transported during the 4-months winter period (December-March). Compared to the cumulative lateral down-canyon directed near bottom flux of POC during the April to May spring bloom (5.1 kg m^−2^) and June to mid-July (≈5.5 kg m^−2^), each of which lasted for two months, we clearly demonstrate the importance of winter fluxes in the transfer of POC to the deep sea.

**Table 2:** Cumulative lateral fluxes of nearbed chlorophyll and derived POC fluxes (from MODIS- SeaDAS) for the period of investigation over monthly periods at the IOV site (Wally).

Month	Down-Canyon Net Chl Flux (g m^−2^ month^−1^)	Down-Canyon Net POC flux (g m^−2^ month^−1^)
November 2010	0.41	75.86
December 2010	11.26	2071.84
January 2011	16.85	3098.51
February 2011	14.74	2709.91
March 2011	8.19	1505.80
April 2011	−0.87	−159.95
May 2011	28.56	5252.04
	Cumulative flux	(g m^−2^) after several months
Sum Nov – May (total 7 months)	79.14	14554.01
Sum Dec – March (total 4 months)	79.60	9386.06
Sum Apr – May (total 2 months)	27.69	5092.08

Note that data gaps in currents at the site (n = 1014 hrs in Nov., Dec. of 5088 hr record) were filled with currents from instruments located nearby within the canyon bottom (Pod3).

We consider it likely that comparable processes operate at all canyons in the northeast Pacific, a region of continental slope with ≈20% canyon cover^9, 33^. These canyons act as large transfer conduits for POC export to depth, with the flux potentially further increased by transfer effects associated with the many gullies commonly connecting to them^34^. Short phytoplankton pulses during winter are also evident in MODIS data collected from other regions of the globe, including the productive subarctic North Atlantic waters^20^. The calculated POC fluxes into the ocean’s bathypelagic zone, by far the single largest inventory of organic carbon on Earth^1^, would be much increased if flux-measurements during winter, as presented in this current study, were to be incorporated. In the canyons of the subarctic northeast Pacific these POC fluxes are further enhanced by the occurrence of iron during winter production^35^, and POC is protected from faster degradation during the many resuspension loops by aggregate-compaction and carbon-armoring (Mode 1) and transport through the oxygen deficient zone^22, 36^. This underlines the importance of new emphasis on winter processes, including field observations on phytoplankton growth and grazer pressure using cabled observatories and extended modeling studies, with the aim of improving the understanding of the total global export of carbon from surface waters to sites of ultimate burial in the deep sea^29, 37^.

## Methods

Our observations result from a monitoring strategy combining satellite (MODIS), surface ocean weather-buoy (http://www.ec.gc.ca), and multi-sensor time-series data (including video, photographic and acoustic seafloor imaging) of BBL processes using Ocean Networks Canada (ONC) seafloor cabled observatory. ONC’s network consists of five subsea observatory nodes linked by 800 kilometers of powered electro-optic cables, looping across the northern Juan de Fuca tectonic plate^38^ (Fig. 1A).

We concentrated our research on oceanographic processes near a gas hydrate mound in Barkley Canyon at 870-m depth, using an Internet-Operated Vehicle (IOV)^13^. The IOV at time of study was connected to a junction box with a 70-m long tethered power/Ethernet cable and is remotely controlled from any computer *via* a specific driving web interface. The IOV moves by caterpillar propulsion, and surveys up to 15000 m^2^ of sediment surface around the gas hydrate mound in high-frequency (≥1 Hz data flow), in real-time, *via* remote control. The caterpillar drive creates a footprint on the seafloor of 0.35 m^2^ with a weight of ≈10 g/cm^2^. All crawler video-transect analyses were conducted along the same transect line to reduce sediment disturbance. Sensor data recorded during video-transects were discarded to avoid artifacts associated with mechanical resuspension of seafloor sediments The sensors attached to the crawler comprised of a CTD (Conductivity, Temperature, Depth) at 20 cm height above the bottom (h.a.b.) (ADM electronics, Germany), fluorescence at 30 cm h.a.b. for the determination of chlorophyll (forward looking sensor Seapoint, USA), optical backscatter at 50 cm h.a.b. (Seapoint, USA), and flow velocity and direction sensor at 100 cm h.a.b. (HS Engineers, Germany). The calibration of the Seapoint fluorometer was performed using microalgae commonly found at the study site and subsequent calculation of the correlation between sensor data and the concentration of algae determined by a Turner Design-700 Laboratory Fluorometer^39^. Biofouling started to influence the chlorophyll*-a* signal by mid July. Before that time, the chlorophyll concentrations decreased to near zero regularly, which indicates no biofouling. The data sampling rate was ≥1 Hz. Fluxes of chlorophyll were calculated using *in situ* flow data from the flow meter and chlorophyll data from the Seapoint sensor with both sensors located on the IOV. Flow conditions in the canyon were determined both at the flanks and in the canyon axis via additional ADCPs, 200 m and 1000 m away. Despite the complex topography of the canyon, flow velocity measurements with these up- and down-looking ADCPs near seafloor at both the canyon axis and on the flanks corresponded with the flow meter on the IOV, all showing regularly occurring downslope-directed flow.

In order to evaluate the benthopelagic coupling of chlorophyll-a from the sea surface downward to the BBL (BBL-Chl), daily averaged satellite data of the U.S. MODerate resolution Imaging Spectrometer (MODIS) were analyzed. MODIS collects surface ocean chlorophyll*-a* data for the full range of seasonal conditions^40^. These satellite data are acquired at daily intervals with a 1 km spatial resolution. In order to minimize data-gaps due to cloud cover, all available daily MODIS chlorophyll data from the continental margin off Vancouver Island (British Columbia coast) described as “Regime 3”^14^ were used. Regime 3 encompasses the waters to the south and west of Vancouver Island and the Washington coast and includes four zones with similar seasonal variations of surface chlorophyll concentrations, with regularly occurring peaks between January and March of each year. Our data were processed in SeaDAS 7.2. (http://seadas.gsfc.nasa.gov) for subsequent analyses. Missing data due to cloud cover in the Regime 3 area were interpolated using Matlab. Calculated weekly averages of our results were compared with the MODIS, 8-day composites provided by NASA for the same period. The Aqua MODIS satellite observes water leaving radiance from the surface ocean. POC is estimated through empirical relationship derived from blue-to-green band ratios of remote sensing reflectance and *in situ* measurements of POC^31^. Estimations of POC from MODIS satellite-based observations were derived via SeaDAS from monthly correlations of chlorophyll/POC in surface waters. Daily averaged data on wave height, wind direction and magnitude were assessed from two coastal buoys (buoy 46206, La Perouse; buoy 46132, South Brooks). We used the Bakun Upwelling Index as indicator of the strength and variations in local coastal upwelling at 48°N, 125°W (southwest of Vancouver Island). We averaged hourly and daily data in order to determine possible correlations between the surface ocean attributes and BBL processes using a non-parametric Kendall’s Tau test, which accounts for temporal autocorrelation and is frequently used for non-normal data distributions typical for hydrological data^41^. Time-series analyses of both time and frequency domains were carried out to evaluate the impact of tidal- and storm induced sedimentation of phytoplankton and previously documented types of oscillatory flow in the northeast Pacific^42^.

Waveform analysis^43^ for the parameters flow (velocity), shear-stress, BBL-Chl, BBl-Chl-Flux, temperature, pressure, turbidity, downslope directed flow (averaged at a frequency of 60 min.) was carried out for each month independently, and separately, in order to assess the phase (i.e. peak timing) of any inherent diel (i.e. 24-h based) cycle matching the local, mixed diurnal, and semidiurnal tidal regime, as potentially influencing the benthopelagic coupling via chlorophyll. The daily datasets were subdivided into 24-h subsets. In this manner, a consensus curve (i.e. the waveform) was obtained and plotted over a standard 24-h period. The peak temporal amplitude was then estimated by setting a threshold to be placed onto the waveform plot and to be used to discriminate values above it. This threshold was determined as the Midline Estimated Statistic of Rhythm (MESOR), used in chronobiology to precisely define waveform peaks’ temporal amplitude according to an onset and an offset (i.e. respectively the first and the last value above the MESOR^44^). We considered as significant peaks, only those average increases made by a minimum of three consecutive values above MESOR.

The temporal relationship among the phases of all oceanographic parameters was visualized by an integrated waveform chart either as a proxy of co-variation or cause-effect relationships^45^. In this chart, the temporal amplitude of each peak was transformed into a horizontal continuous line whose onset and offset can be visually related with the onsets and offsets of all others parameters. Since waveform analysis was conducted throughout each month, we plotted integrated charts accordingly.

A Canonical Correlation Analysis^46^ (CCA) was performed in a Time-Lagged (TL) fashion (from 12 to 72 hrs, with a step of 12 hrs, related to the results from MODIS data and to the tidal cycle), to assess the shift in chlorophyll concentration measured at the IOV (BBL-Chl) as a response variable against other environmental parameters. The parameters could be putative drivers of the measured value change and include: temperature and flow velocity as measured by the IOV, flow velocity from the Canyon Axis, meteorological- and wave data as measured by South Brooks Buoy and La Peruse Buoy. Briefly, the aim of using CCA is to find the best linear combination between two multivariate datasets that maximizes the correlation coefficient between them^47^. Canonical coefficients, which represent the degree of correlation between each biological response variable and environmental variables dataset, were reported together with their significance (Bartlett’s χ^2^ test). This test assumes the null hypothesis of no correlation between the two sets. The time lag represents the gap among the BBL-Chl and the aforementioned environmental variables shifted by n-hours before (e.g. for a time lag = 24 hrs)^46^.

In addition to carrying out environmental sampling, the IOV collected video imagery data during the first three months of 2011, to capture any responses by the benthic fauna to the chlorophyll pulses to the BBL and to other environmental variability. Additionally, acoustic imaging monitoring of benthic megafauna in the survey region was conducted with two rotary sonars during the entire period overlapping with the IOV monitoring (Nov 2010-July 2011). For the interested reader the methods and results from these investigations, which corroborate our hypothesis of pelagic-benthic coupling in Barkley Canyon following winter-triggered POC fluxes to the canyon seafloor, are described in the Supplementary Material.

### Data

Original data and videos from rotary-sonars installed near the study site can be observed along with other data from this project at the ONC data bank (http://dmas.uvic.ca/home).

All post processed data sets such as the MODIS satellite data, surface weather buoy data, and flux calculations, are available online through the Dryad data repository.

## Electronic supplementary material

Supplementary Information

## References

[1] Honjo S (2014). Understanding the role of the Biological Pump in the Global Carbon Cycle: An imperative for Ocean Science. Oceanography.

[2] Muller‐Karger, F. E. *et al*. The importance of continental margins in the global carbon cycle. *Geophysical Research Letters*, 32(1) (2005).

[3] Biscaye PE, Anderson RF (1994). *Fluxes of particulate matter on the slope of the southern Middle Atlantic Bight: SEEP-II*. Deep-Sea Research II.

[4] Wollast R, Chou L (2001). *Ocean margin exchange in the northern Gulf of Biscay: OMEX I*. *An Introduction*. Deep Sea Research Part II.

[5] Weaver PP, Canals M, Trincardi F (2006). Eurostrataform. Marine Geology.

[6] Allen SE, D de Madron X (2009). *A review of the role of submarine canyons in deep-ocean exchange with the shelf*. Ocean Science.

[7] De Leo, F. C., Smith, C. R., Rowden, A. A., Bowden, D. A. & Clark, M. R. Submarine canyons: hotspots of benthic biomass and productivity in the deep sea. Proc. Royal Society of London B: Biological Sciences, rspb20100462 (2010). 10.1098/rspb.2010.0462 PMC2981985 20444722

[8] Canals M (2006). Flushing submarine canyons. Nature.

[9] Ladd C, Stabeno P, Cokelet ED (2005). *A note on cross-shelf exchange in the northern Gulf of Alaska*. Deep Sea Research Part II.

[10] Guidi L (2009). Effects of phytoplankton community on production, size, and export of large aggregates: A world‐ocean analysis. Limnology and Oceanography.

[11] Lomas MW (2009). Biogeochemical responses to late-winter storms in the Sargasso Sea. IV. Rapid succession of major phytoplankton groups. Deep Sea Research Part I.

[12] Bishop JK, Fong MB, Wood TJ (2016). *Robotic observations of high wintertime carbon export in California coastal waters*. Biogeosciences.

[13] Thomsen, L. *et al*. Ocean circulation promotes methane release from gas hydrate outcrops at the NEPTUNE Canada Barkley Canyon node. Geophys. Res. Lett. **39**(16) (2012).

[14] Jackson JM, Thomson RE, Brown LN, Willis PG, Borstad GA (2015). *Satellite Chlorophyll off the British Columbia Coast*, *1997-2010*. J. Geophysical Research.

[15] NASA Goddard Space Flight Center, Ocean Ecology Laboratory, Ocean Biology Processing Group. Moderate-resolution Imaging Spectroradiometer (MODIS) Terra Aqua Chlorophyll Data; NASA OB.DAAC, Greenbelt, MD, USA. https://oceancolor.gsfc.nasa.gov/data/aqua/. Accessed on 06/2016 https://oceancolor.gsfc.nasa.gov. Maintained by NASA Ocean Biology Distributed Active Archive Center (OB.DAAC), Goddard Space Flight Center, Greenbelt MD.

[16] Agusti, S. *et al*. Ubiquitous healthy diatoms in the deep sea confirm deep carbon injection by the biological pump. *Nature communications* **6** (2015). 10.1038/ncomms8608 PMC4510647 26158221

[17] Ferrari, R., Merrifield, S. T., & Taylor, J. R. Shutdown of convection triggers increase of surface Chlorophyll. *J*. *Mar*. *Syst*., doi: 10.1016/j.jmarsys.2014.02.009 (2014).

[18] Lindemann C, St John MA (2014). A seasonal diary of phytoplankton in the North Atlantic. Frontiers in Marine Science.

[19] Turner JT (2015). Zooplankton fecal pellets, marine snow, phytodetritus and the ocean’s biological pump. Progress in Oceanography.

[20] Behrenfeld MJ, Boss ES (2014). Resurrecting the ecological underpinnings of ocean plankton blooms. Ann. Rev. Mar. Sci..

[21] Townsend DW, Keller MD, Sieracki ME, Ackleson SG (1992). *Spring phytoplankton blooms in the absence of vertical water column stratification*. Nature..

[22] Thomsen L, McCave IN (2000). *Aggregation processes in the benthic boundary layer at the Celtic Sea continental margin*. Deep-Sea. Research I.

[23] Allen SE, Hickey BM (2010). *Dynamics of advection-driven upwelling over a shelf break submarine canyon*. J. Geophys. Res..

[24] Thomson RE, Mihaly SF, Kulikov EA (2007). *Estuarine versus transient flow regimes in Juan de Fuca Strait*. J. Geophys. Res..

[25] Hickey BM (1997). Response of a narrow submarine canyon to strong wind forcing. J. Physical Oceanography.

[26] Connolly TP, Hickey BM (2014). Regional impact of submarine canyons during seasonal upwelling. J. Geophys. Res..

[27] Waite AM (2016). The wineglass effect shapes particle export to the deep ocean in mesoscale eddies. Geophys. Res. Letters.

[28] Chang AS (2013). Annual record of particle fluxes, geochemistry and diatoms in Effingham Inlet, British Columbia, Canada, and the impact of the 1999 La Niña event. Marine Geology.

[29] Banse K (1994). 1994. Grazing and zooplankton production as key controls of phytoplankton production in the open ocean. Oceanography.

[30] Guidi L (2007). Vertical distribution of aggregates (>110 µm) and mesoscale activity in the northeastern Atlantic: Effects on the deep vertical export of surface carbon. Limnology and Oceanography.

[31] Stramska M (2014). Particulate organic carbon in the surface waters of the North Atlantic: spatial and temporal variability based on satellite ocean color. Int. J. Remote Sensing.

[32] Sun M, Aller RC, Lee C (1991). *Early diagenesis of Chlorophyll-a in Long Island Sound sediments: A measure of carbon flux and particle reworking*. J. Marine Research.

[33] Hickey, B. M. of referencing in Coastal submarine canyons (ed. Muller, P. & D. Henderson), (Proc. University of Hawaii ‘Aha Huliko’a Workshop on Flow Topography Interactions, Honolulu, Hawaii, SOEST Special Publication, 1995).

[34] Gales JA (2013). Arctic and Antarctic submarine gullies—A comparison of high latitude continental margins. Geomorphology.

[35] Lam, P. J. *et al*. Wintertime phytoplankton bloom in the subarctic Pacific supported by continental margin iron. Global Biogeochemical Cycles, **20**(1) (2006).

[36] Keil RG, Neibauer JA, Devol AH (2016). *A multiproxy approach to understanding the” enhanced” flux of organic matter through the oxygen-deficient waters of the Arabian Sea*. Biogeosciences.

[37] Alford MH, MacCready P (2014). *Flow and mixing in Juan de Fuca Canyon*, *Washington*. Geophys. Res. Lett..

[38] Barnes, C. R., Best, M. M., Pautet, L., & Pirenne, B. *Understanding Earth–Ocean processes using real-time data from NEPTUNE*, *Canada’s widely distributed sensor networks*, *northeast Pacific*. Geoscience Canada **38**(1) (2011).

[39] Yentsch CS, Menzel DW (1963). *A method for the determination of phytoplankton Chlorophyll and phaeophytin by fluorescence*. Deep Sea Research.

[40] Gower JF, Brown L, Borstad GA (2004). *Observation of Chlorophyll fluorescence in west coast waters of Canada using the MODIS satellite sensor*. Canadian Journal of Remote Sensing.

[41] Hirsch RM, Slack JR (1984). *A nonparametric trend test for seasonal data with serial dependence*. Water Resources Research.

[42] Connolly TP, Hickey BM (2014). *Regional impact of submarine canyons during seasonal upwelling*. J. Geophy. Res..

[43] Aguzzi J (2009). Chronobiology of deep-water decapod crustaceans on continental margins. Advances in marine biology.

[44] Refinetti R, Cornélissen G, Halberg F (2007). *Procedures for numerical analysis of circadian rhythms*. Biological Rhythm Research.

[45] Aguzzi J (2012). Challenges to the assessment of benthic populations and biodiversity as a result of rhythmic behaviour: Video solutions from cabled observatories. Oceanography and Marine Biology-An Annual Review.

[46] Sgarbossa A (2014). Colorimetric patterns of wood pellets and their relations with quality and energy parameters. Fuel.

[47] Sherry A, Henson RK (2005). *Conducting and interpreting canonical correlation analysis in personality research: A user-friendly primer*. J. Personality Assessment.

