# Supplementary material for: The Oceanic Biological Pump: Rapid carbon transfer to depth at Continental Margins during Winter
Source: Sci Rep. 2017 Sep 7;7:10763. doi: 10.1038/s41598-017-11075-6 (PMC5589845; doi:10.1038/s41598-017-11075-6)
Supplement: Supplementary file 1 — Supplementary Information [file 41598_2017_11075_MOESM1_ESM.pdf]

Title:

The Oceanic Biological Pump: Rapid carbon transfer to depth at Continental Margins during Winter

Authors:

*Laurenz Thomsen[1], Jacopo Aguzzi[2], Corrado Costa[3], Fabio De Leo[4], Andrea Ogston[5], Autun Purser[6]

Affiliations:

[1]*Jacobs University, Bremen, 28759, Germany*, [2]Instituto de Ciencias del Mar (ICM-CSIC), Barcelona, 08003, Spain, [3]Consiglio per la ricerca in agricoltura e l'analisi dell'economina agrarian (CREA-IT), Monterotondo, 00016, Italy, [4]Ocean Networks Canada and Department of Biology, University of Victoria, BC, V8W 2Y2, Canada, [5]School of Oceanography, University of Washington, Seattle, 98195, USA, [6]Alfred Wegener Institute for Polar Research, Bremerhaven, 27570, Germany.

* Correspondence to: l.thomsen@jacobs-university.de

Supplementary materials

A. Bentho-pelagic coupling in Barkley Canyon: the benthic fauna response to inputs of particulate organic matter derived from winter and spring phytoplankton blooms.

(1) Photo mosaics assembled using the IOV's video footage

During the first three months of 2011, a forward facing web-camera mounted in the IOV (640 x 480 pixel resolution) was used to repeatedly image ≈ 20 m^2, area of a gas hydrate mound in intersection with the surrounding seafloor. Images were taken by using the pan and tilt functionality of the camera to take overlapping images ~10 degrees (vertically and horizontally) apart from each other, whilst the IOV moved slowly along the mound. This procedure collected up to 90 images of the region of interest with each survey, which were mosaicked together using Photoshop CS5 (i.e., the 'collage' mosaicking algorithm used with the 'photomerge' function). (*)Examples of individual images and produced mosaics are given in Supplementary Figure 1.

The surveyed area was dominated mostly by the cold-seep conspicuous species, the vesicomyid clam *Vesycomya (Calyptogena) pacifica*. Non-seep endemic species such as the grooved tanner crab, *Chalionoecetes tanneri*, the gastropod *Neptunea* spp., and the sablefish *Anoplopoma fimbria*, were also present in moderate to lower abundances.

The photo mosaics were then uploaded into the post-processing ImageJ software[1], where image analysis of the benthic fauna was carried out. The number of open and closed vesycomid clams, buried and exposed tanner crabs, and the total numbers of gastropods, were logged for each photo mosaic.

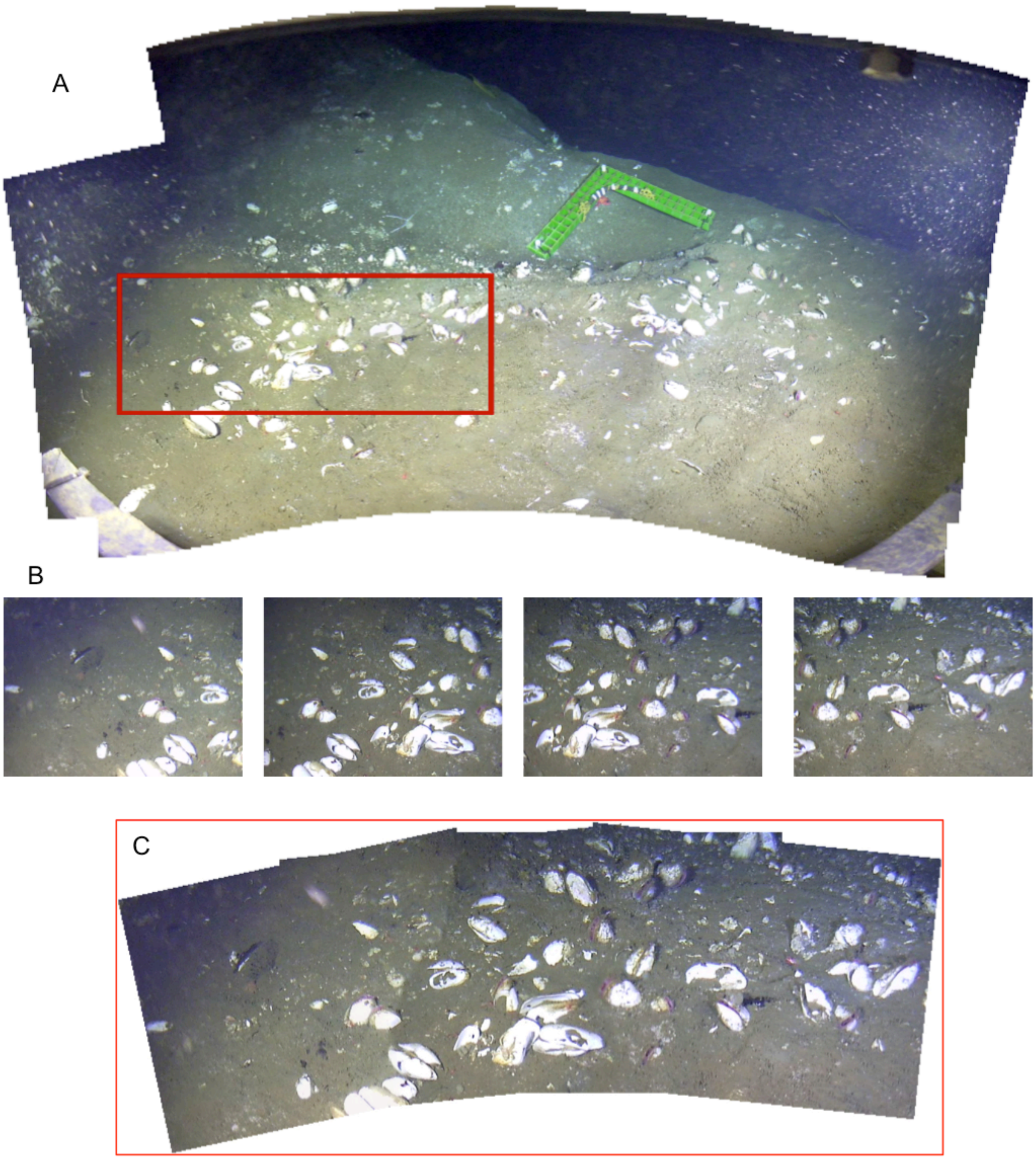

Supplementary Figure 1 – Example of a photo-mosaic assembled based on still photographs taken with the IOV video camera at one location. A, full mosaic of the section of a hydrate mound / seafloor intersection analyzed in the current study for fauna abundance / behavior. B, individual and overlapping images collected at 10° intervals in the horizontal plane while panning the camera. C, A close up of the four photographs in B mosaiced together, showing the level of detail achievable via the mosaicing process.

(2) Canonical Correspondence Analysis: Environmental variables vs benthic fauna relative abundance and activity

The relative % abundances of open and shut clams, and buried and exposed crabs computed from the previous analyses of the photomosaics were used in a Canonical Correspondence Analysis (see Methods in the main text), in order to determine if benthic species' abundances and activity responded to the BBL-chlorophyll inventories and flux, bottom temperature, pressure, turbidity, sheer stress and bottom current speeds within Barkley canyon's BBL.

The CCA combining fauna count data from the IOV photomosaics and environmental variables measured by the vehicle (Table S1) revealed that juvenile tanner crabs lay passive and half-buried in sediments despite elevated BBL-Chl (-0.71) and when shear-stress was elevated (-0.79, Mode 1). However, they were actively deposit-feeding in surface sediments when both BBL-Chl and bottom-water-temperatures were elevated (0.84/0.38, Mode 2). These corroborated similar observations from an earlier study, which hypothesized that this species in particular occurs in higher abundances under low shear stress conditions, avoiding sediment resuspension during foraging[2]. A similar behavior was recorded for deposit feeding gastropods, which were most abundant during times of low flow and elevated BBL-Chl (0.69/0.52, Mode 2) in February. The typical cold-seep species (vesicomyid clams) showed no correlation in its activity to elevated BBL-Chl, but were actively pumping seawater (opening/closing valves) during times of elevated flow (0.5).

Table S1. Canonical Correlation Analyses (CCA) from IOV photomosaics and environmental variables showing relationships (* significant, $p<0.05$) with the cyclic fluctuation of the chlorophyll signal in the BBL (BBL-Chlorophyll)

**Abundance of juvenile tanner crabs**			Overall CCA Coeff.
Low sheer stress	0.79	*	0.54
Low temperature	0.62	*	0.54
Low BBL-Chlorophyll	0.66	*	0.54
**Juvenile tanner crabs active deposit feeding**			
Elevated temperature	0.38		0.27
Elevated BBL-Chlorophyll	0.84		0.27
**Number of open vesicomyid clams**			
High flow velocity	0.5	*	0.57
Low temperature	0.63	*	0.57
Low BBL-Chlorophyll	0.60	*	0.57
**Number of closed vesicomyid clams**			
Low flow velocity			
Low temperature	0.63		0.42

Low BBL-Chlorophyll	0.60		0.42
**Abundance of Neptunea snails**			
Low flow velocity	0.69		0.40
Elevated BBL-Chlorophyll	0.52		

(3) Multi-frequency rotary sonars

Our observations of the benthic fauna using the IOV video/still camera (i.e., from the photo mosaics) were further supported and complemented by data analyzed from two imaging rotary sonars installed in two additional locations a few hundred of meters away from the Barkley Hydrates site, at the Canyon Wall and Canyon Axis sites (Figure 1B, main text). The 675 kHz Kongsberg imaging rotary sonars have a 10 m range in radius, sampling a ~50m^2 area of the seafloor while performing 360° sweeps (beam width of ~15 degrees). The device configuration, sampling schedules and data are publicly available through ONC's *'Oceans 2.0'* data management and archiving system (http://dmas.uvic.ca/DeviceListing?DeviceId=11301; http://dmas.uvic.ca/DeviceListing?DeviceId=11301).

The sonars at time of study were set on automatic schedules performing 10-12 sweeps at hourly intervals. We used data only from the first hourly sweep from each duty cycle, generating backscatter amplitude imaging plots, which were then assembled into time-lapse movies, recorded at a 300 millisecond frame rate (note: all generated time-lapse videos are provided as supplementary videos, SV1-8). We have extracted data from a total of four time periods (Supplementary Figures, SF2,3), and from the Canyon Wall and Canyon Axis locations (Fig1B, main text), therefore totalizing 8 different data sets that matched the most relevant periods of our seafloor (and BBL) measurements and observations. The individual sonar time-series data sets were comprised of hourly sweeps during 10-12 days: i) Nov 15-27 2010 (Canyon wall), Nov 19-29 (Canyon Axis), when no signal of BBL-Chlorophyll (i.e., fresh phytodetritus) was evident; ii) Feb 7-19, 2011 (Canyon Wall), Feb 7-18, 2011 (Canyon Axis), during maximum winter chlorophyll peaks at the BBL; iii) April 11-22, 2011 (Canyon Wall), 11-23 April (Canyon Axis), during low to moderate chlorophyll fluxes to the BBL; iv) May 2-13, 2011 (Canyon Wall), May 2-14 (Canyon Axis), during maximum BBL-Chlorophyll signals when comparing with the entire studied period (SF2-3).

During mid-to-late November 2010, when no relevant phytodetrital signal was detected within the BBL, the sediment surface revealed very little benthic activity (Supplementary Figures, SF2,3; Supplementary videos SV1,2). This changed in early-to-mid February 2011, when maximum winter chlorophyll peaks were detected at the BBL, with concomitant increase in epibenthic megafauna abundance and activity mostly by juvenile tanner crabs, and to a lesser extent by sablefish (SF2,3; SV3,4). During mid-to-late April 2011, when surface spring bloom was fully developed but with no strong BBL-Chlorophyll signals yet apparent, megafaunal abundance and activity was mostly attributed to sablefish (SF2,3; SV5,6). In early-to-mid May maximum benthic activity occurred simultaneously and after post-spring bloom POC flux reached the BBL. During this period, the BBL-Chlorophyll signals reached the highest values

when compared with the entire period of observations and once again juvenile tanner crabs dominated the benthic fauna (SF3, SV7-8).

In a more recent observation, a mass migration of juvenile tanner crabs (with carapace lengths smaller than 88 mm for females and 112 mm for males[3]) occurred during mid winter (January of 2015) in Barkley Canyon, which would coincide with these pulsed winter POC fluxes to the BBL. While mass migrations are common among many crustacean species, no previous studies have specifically addressed the main ecological factors explaining these migrations in tanner crabs. Nevertheless, several hypotheses have been postulated to explain these tanner crab mass migration events, which include: (1) an unidirectional ontogenetic movement where juvenile crabs move towards upper slope (via canyon) into optimal adult foraging habitats; (2) aggregation and movement that prevents juveniles from being predated, particularly during molting season (i.e., predator swamping hypothesis[4]); or (3) massive trophic focusing due to a rapid population response to increased prey availability (i.e., infaunal macrobenthos) after organic enrichment of surface sediments by fresh phytodetritus. While these hypotheses remain largely untested, the general responses by the benthic fauna we observed during our study, where no mass migration events were observed, help to support the trophic-focusing hypothesis. Hence, a topographically constraining feature such as Barkley Canyon, may channel particulate organic carbon in the form of phytodetritus, which settles in the seafloor during periods of low BBL current flow, becoming readily available for the benthic infauna and epifauna.

Future studies combining the use of long-term monitoring using ONC's cable observatory assets and data from surface ocean productivity and downward particulate organic carbon fluxes, will help to better elucidate the timing and spatial extent of the pelagic-benthic coupling in Barkley Canyon.

*Video of showing a tanner crab mass migration event in Barkley Canyon Axis (Feb 12, 2015 18:00 UTC):

(http://dmas.uvic.ca/SeaTube?resourceTypeId=1000&resourceId=23152&diveId=15419055&time=2015-01-12T18:00:19.000Z)

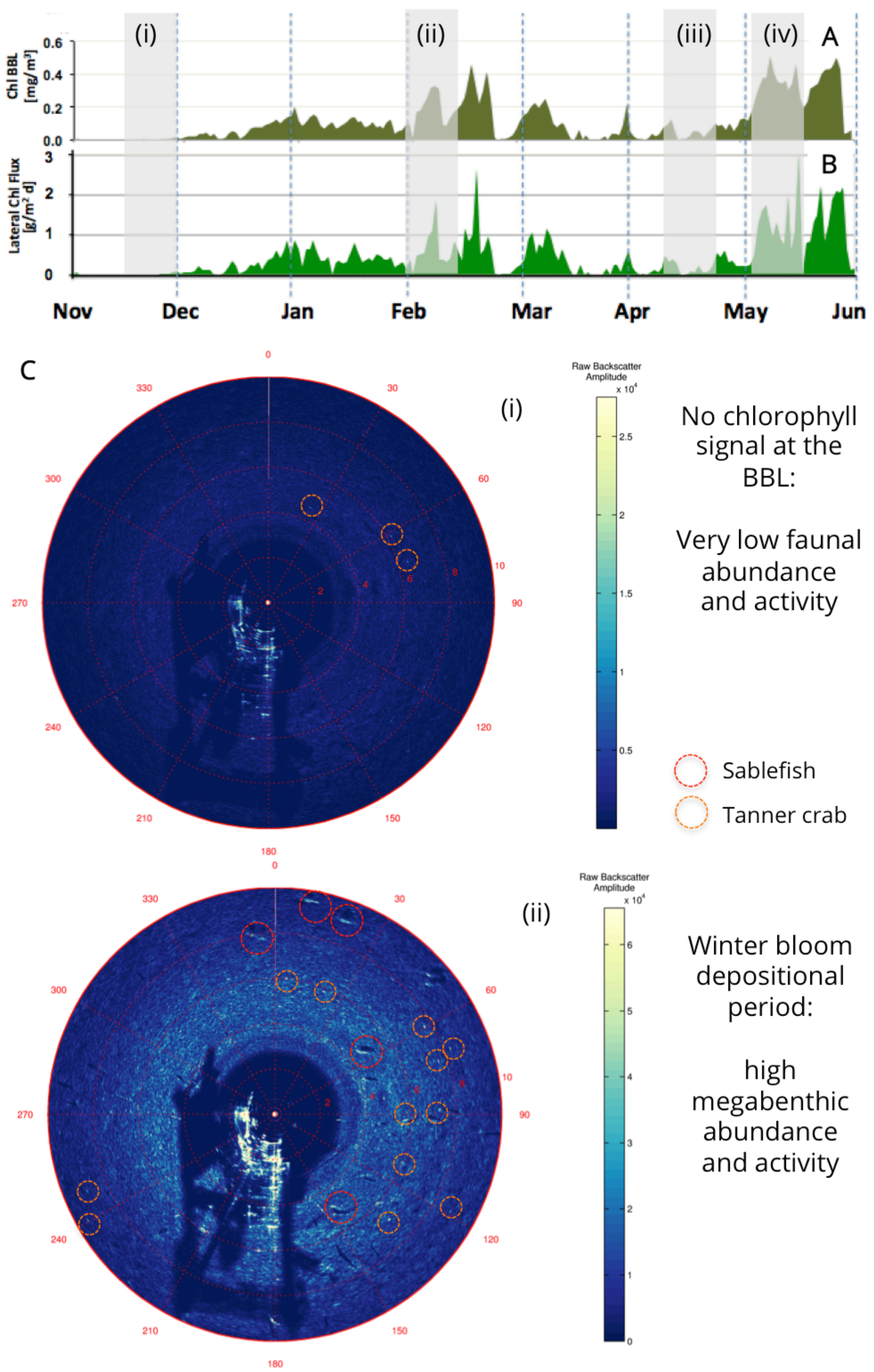

Supplementary Figure 2. Upper panels modified from Figure 2 in the main text: A, BBL-Chlorophyll concentration (mg/m^3). B, BBL lateral chlorophyll flux (mg/m^3 day^{-1}). Lower panels: C, examples of 675 kHz rotary sonar sweeping scans showing benthic species during mid-to-late November 2010 (i), and early-to-mid February 2011 (ii). Note that sonar range radius is 10 m.

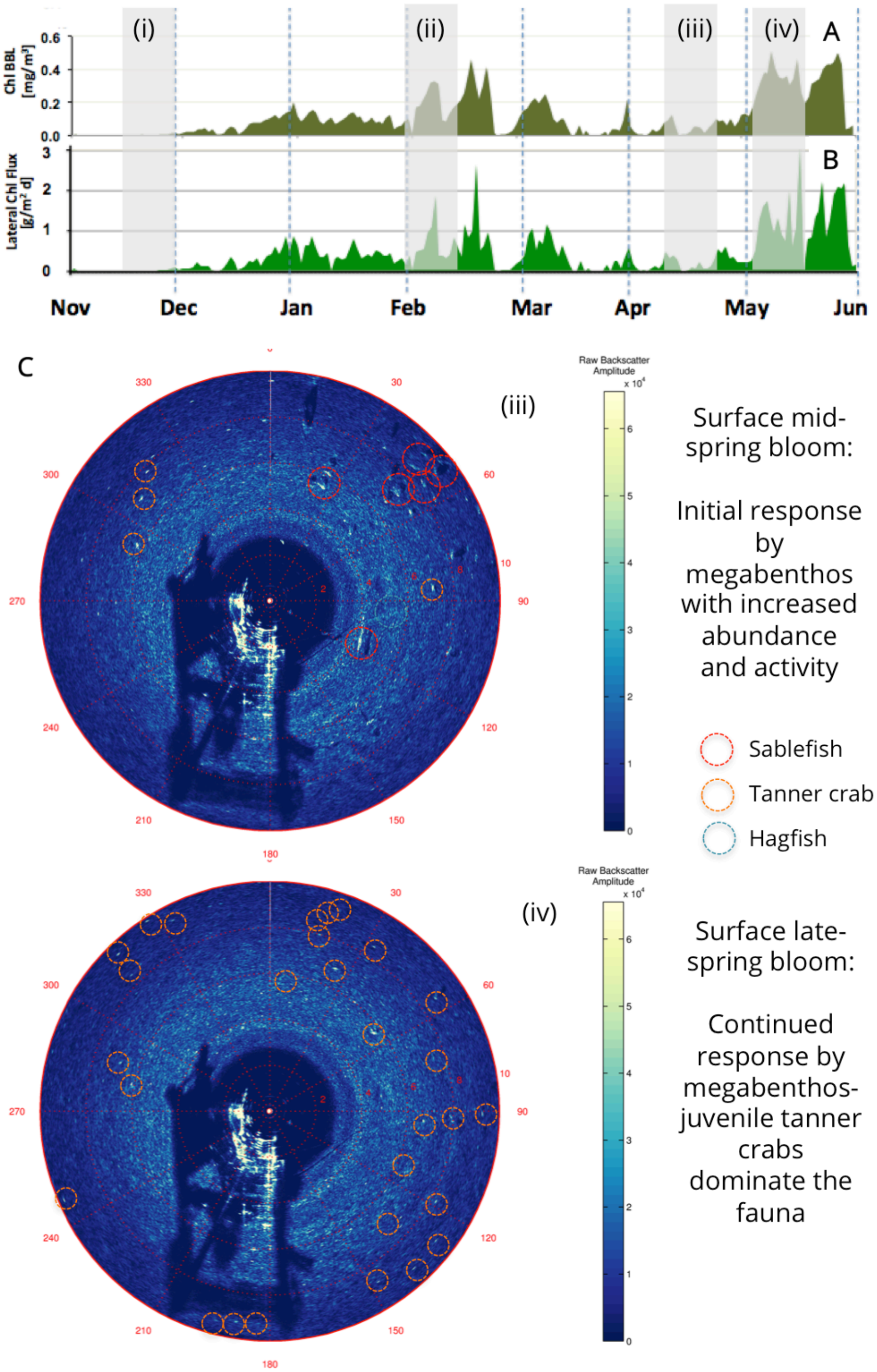

Supplementary Figure 3. Upper panels modified from Figure 2 in the main text: A, BBL-Chlorophyll concentration (mg/m^3). B, BBL lateral chlorophyll flux (mg/m^3 day^{-1}). Lower panels: C, examples of 675 kHz rotary sonar sweeping scans showing benthic species during mid-to-late April 2011 (iii), and early-to-mid May 2011 (iv). Note that sonar range radius is 10 m.

(4) Additional info on down-canyon fluxes

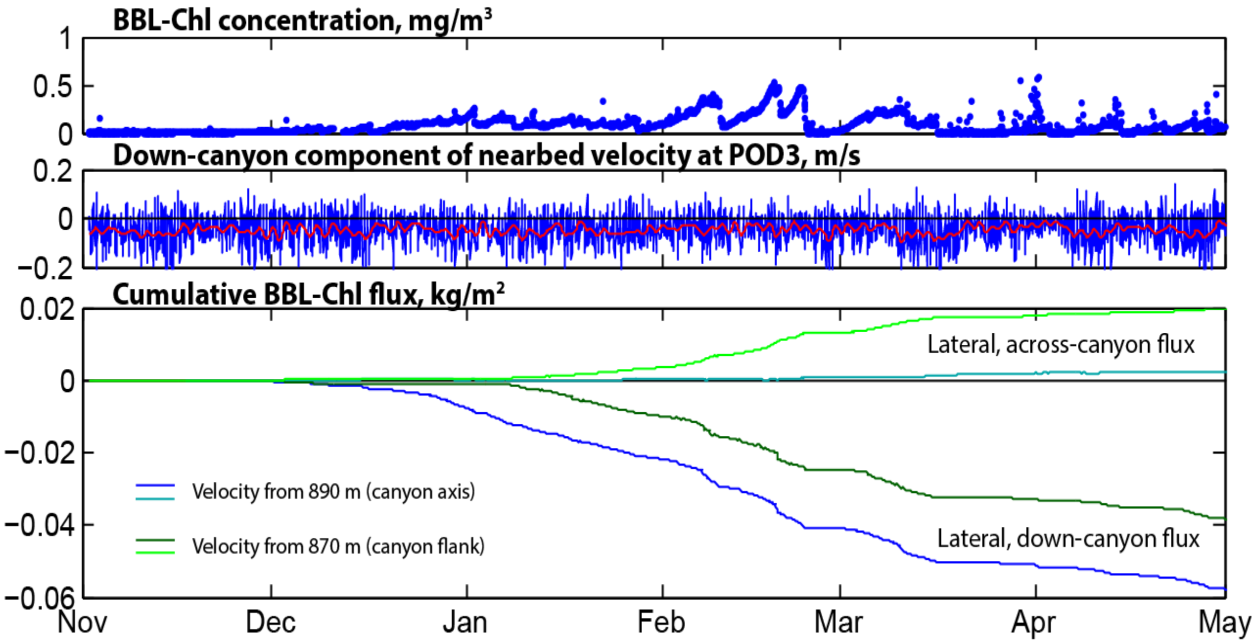

Supplementary Figure 4. Hourly BBL-Chl concentration, velocity in the canyon axis, and cumulative BBL-Chl flux. BBL-Chl was measured at a site on the canyon flank (870 m water depth) associated with complex bathymetry. To represent down-axis BBL-Chl fluxes, the hourly fluxes were estimated using both the currents at the canyon axis (POD3) and at the flank site collocated with the BBL-Chl concentration observations. Note that although magnitudes differ, the patterns of net flux are the same for both analyses, with the greatest periods of net flux occurring associated with the period of small winter blooms at the sea surface (Fig.2, main text). Shown here are hourly currents (blue) and low-pass filtered currents (38 hr cut-off; red) at the canyon axis.

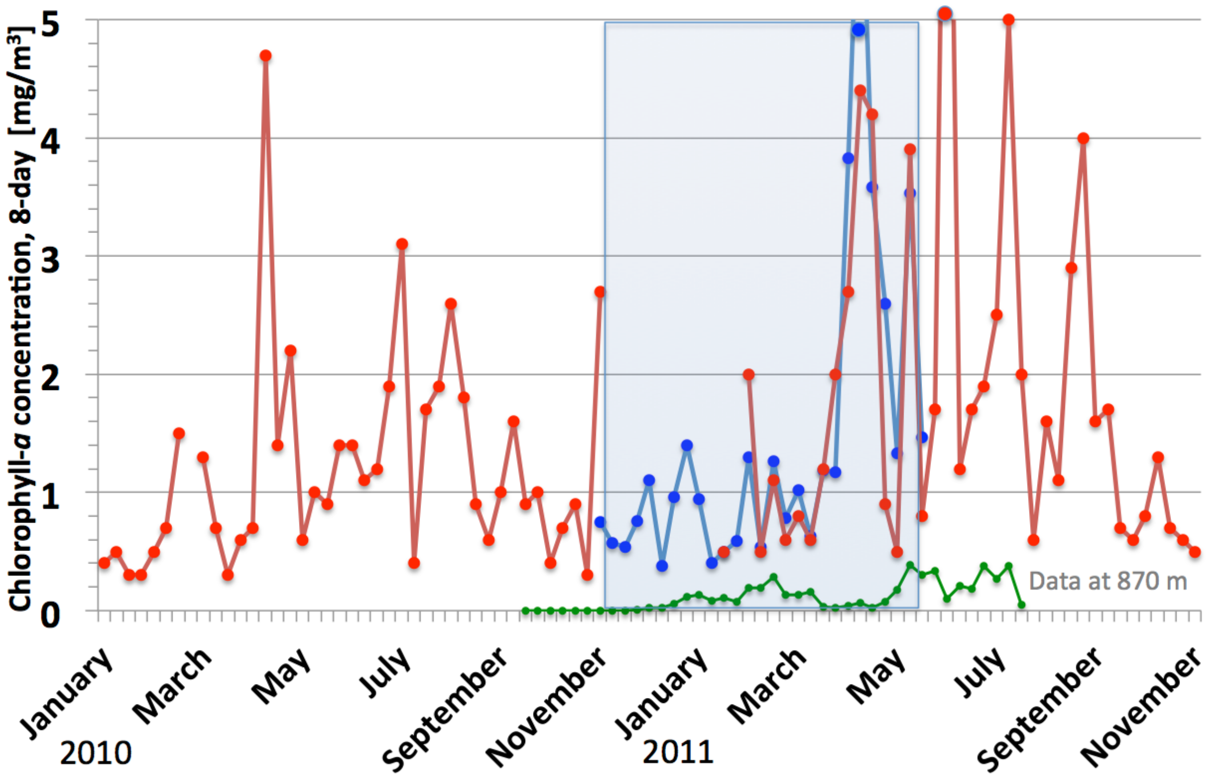

Supplementary Figure 5. Comparison of 8-day averaged chlorophyll-*a* time series from MODIS[15] (red dots: provided by NASA; blue dots: averaged from own analyses as part of this study), and sensor data of chlorophyll in bottom waters (BBL-Chl, green dots: averaged from a Seapoint fluorometer at 0.3 m above seafloor) for the area described as "Regime 3"[14] during 2010/2011. Our analyses for the period October 2010 to June 2011 showed good agreement with the 8-day composites from NASA (r^2=0.76). Measurements of chlorophyll-*a* in bottom waters of Barkley Canyon (green line) at 870-m water depth using the IOV highlight the occurrence of a considerable flux of chlorophyll-*a* during that time. It is hypothesized to link the low chlorophyll concentrations in the BBL between September and December to stratification, when maximum grazer pressure and elevated SST prevented significant carbon fluxes.

Additional References

1. Rasband, W.S., 2009 IMAGE J. US National Institutes of Health, Bethesda, MD. See: http://rsb.info.nih.gov/ij/〉 .

2. Doya, C., Aguzzi. J., Pardo, M., Matabos, M. Company, J.B., Costa, C., Mihály, S., Canals, M., 2014. Diel behavior rhythms in sablefish (*Anoplopoma fimbria*) and other benthic species, as recorded by the deep-sea cabled observatory in Barkley Canyon (NEPTUNE-Canada). Journal of Marine Systems 130, 69-78.

3. Workman, G.D., Boutillier, J.A., Phillips, A.C., Gillespie, G.E., Park, W.G., Clark, D., Pennell, B., 2001. Results of a Bottom Trawl Survey of Grooved Tanner Crab, Chionoecetes tanneri Rathbun, Stocks off the Coast of Vancouver Island, July 21 - Aug 3, 1999. Can. Manuscr. Rep. Fish. Aquat. Sci. 2568. 79 p.

4. Corgos, A., Sánchez N., Freire, J., 2010. Dynamics of the small-scale spatial structure of a population of the spider crab *Maja brachydactyla* (Decapoda: Majidae).
